# Is there a change in the appropriateness of admission after patients were admitted? Evidence from four county hospitals in rural China

**DOI:** 10.3389/fpubh.2023.1106499

**Published:** 2023-05-25

**Authors:** Jingjing Chang, Hongxia Gao, Dai Su, Haomiao Li, Yingchun Chen

**Affiliations:** ^1^Wuhan Children's Hospital (Wuhan Maternal and Child Healthcare Hospital), Tongji Medical College, Huazhong University of Science and Technology, Wuhan, China; ^2^School of Medicine and Health Management, Tongji Medical College, Huazhong University of Science and Technology, Wuhan, China; ^3^Research Center for Rural Health Services, Hubei Province Key Research Institute of Humanities and Social Sciences, Wuhan, China; ^4^School of Public Health, Capital Medical University, Beijing, China; ^5^School of Political Science and Public Administration, Wuhan University, Wuhan, China

**Keywords:** inappropriate admissions, appropriateness evaluation protocol, admission records, medical records, the change in appropriateness of admission

## Abstract

**Objective:**

This study aims to investigate the changes in admission appropriateness after patients were admitted and provide a reference for physicians to make admission decisions and for the supervision of medical service behavior by the medical insurance regulatory department.

**Methods:**

Medical records of 4,343 inpatients were obtained based on the largest and most capable public comprehensive hospital in four counties in central and western China for this retrospective study. The binary logistic regression model was employed to examine the determinants of changes in admission appropriateness.

**Results:**

Nearly two-in-thirds (65.39%) of the 3,401 inappropriate admissions changed to appropriate at discharge. Age, type of medical insurance, medical service type, severity of the patient upon admission, and disease category were found to be associated with the changes in the appropriateness of admission. Older patients (OR = 3.658, 95% CI [2.462–5.435]; *P* < 0.001) were more likely to go from “inappropriate” to “appropriate” than younger counterparts. Compared with circulatory diseases, the case evaluated as “appropriate” at discharge was more frequent in the urinary diseases (OR = 1.709, 95% CI [1.019–2.865]; *P* = 0.042) and genital diseases (OR = 2.998, 95% CI [1.737–5.174]; *P* < 0.001), whereas the opposite finding was observed for patients with respiratory diseases (OR = 0.347, 95% CI [0.268–0.451]; *P* < 0.001) and skeletal and muscular diseases (OR = 0.556, 95% CI [0.355–0.873]; *P* = 0.011).

**Conclusions:**

Many disease characteristics gradually emerged after the patient was admitted, thus the appropriateness of admission changed. Physicians and regulators need to take a dynamic view of disease progression and inappropriate admission. Aside from referring to the appropriateness evaluation protocol (AEP), they both should pay attention to individual and disease characteristics to make a comprehensive judgment, and strict control and attention should be paid to the admission of respiratory, skeletal, and muscular diseases.

## 1. Introduction

The average inpatient utilization rate per capita of the world was 0.10, while that in China was 0.14 (1). The inpatient utilization rate in 2008 in China was 8.7% and it increased to 17.5% in 2021 (2). In the past more than 10 years, the inpatient utilization rate has more than doubled in China, with the rapid growth worth noting.

The increase in inpatient utilization leads to inefficient use of health resources and unreasonable increase in total health expenditure (3). The research report on “The reform of medical and health system in China” in 2016, pointed out that health expenditure (% of GDP) in China will increase from 5.6 in 2014 to over 9 in 2035, of which more than 60% is expected to come from the hospitalization services. The average annual growth rate of total healthcare expenditure in China was 15.06% in the last decade (4). As a typical kind of excessive utilization of hospitalization services (5, 6), inappropriate admission has caused an unreasonable increase in health expenditure (7–9). Inappropriate admission refers to unnecessary hospitalization services and it could be alternated by outpatient services. Previous studies showed that the average inappropriate admission rate was 26.5% in township hospitals and 15.2% in county hospitals in China (10, 11). Therefore, controlling inappropriate admissions and avoidable healthcare expenditure has become a major issue for policymakers.

So far, the methods to identify inappropriate admission can be summarized into two aspects according to whether it is based on the level of disease diagnosis or not. The recognition patterns based on disease diagnosis mainly include clinical pathway, RAND expert group evaluations and clinician experience judgment. These methods can make reasonable and accurate judgments with a comprehensive understanding of the disease. However, these methods are largely influenced by the subjective factors of the judges. The recognition patterns not based on disease diagnosis include the appropriateness evaluation protocol (AEP), intensity–severity–discharge criteria and standardized medreview instruments. These explicit non-diagnostic criteria are measurable, objective, reliable and uniform. Several studies indicated that AEP is the most effective tool to evaluate the appropriateness of admission and has high reliability and validity (12–14).

The existing studies mainly focus on evaluating the inappropriate length of stay in hospitals by using AEP and have paid little attention to the issue of “admission”. Jeddian et al. (15) found that the average length of stay in internal and surgical wards is 9.4 to 6.3 days, whereas 8.5% of admissions and 3.4% of hospital stays are inappropriate. Liu et al. (16) assessed the prevalence of inappropriate length of stay in a tertiary hospital in Shanghai, and found that 910 (25.2%) and 1,940 (40.5%) length of stay in hospital were inappropriate in the cardiology and orthopedics departments, respectively. Sánchez-García S et al. (14) deemed that AEP's high-reliability and moderate-validity results regarding clinical judgement make AEP a useful instrument for appropriate hospitalization screening in older adult patients. They also found that the specificity and negative predictive value to detect appropriate admission was >94.0% and >98.0%. In a prior study by V Granados García (17), AEP was used to evaluate the appropriateness of hospital stay of the old-aged patients, and estimate the direct medical costs related to the appropriateness of inpatient admission among the older adult. They found that the average cost of all 509 patients was 34,769 Mexican pesos (SD = 2,869 pesos), which varied by different age groups.

The above studies have confirmed that inappropriate admission does lead to inappropriate length of stay in hospitals and avoidable medical costs, resulting in a waste of health resources. It is urgent to control inappropriate admission, and the prevention of inappropriate admission is vital. Physicians' judgment on whether a patient needs to be admitted is crucial (10). Since there is no unified inpatient indication standard in China, doctors mainly judge whether the patients should be admitted to take the inpatient care by the patient's symptoms at the time of admission and their own medical experience. There often exist three situations. First, the patient has obvious disease symptom and the disease is serious, the doctor can be sure to admit the patient to hospitalization, which is usually appropriate for inpatient admission. Second, the disease features are not obvious but can't be ignored. The patient need to take further medical examination for clarify the disease, so the doctor admit the patient to hospitalization. The appropriateness of these admission is uncertain. Third, the disease symptoms are very mild, the doctor decided that the patient should only receive outpatient treatment. In the second situation, when the patient discharge, there are two possibilities for the appropriateness of admission. First, the disease was found to be serious after examination and diagnosis, thus the admission was appropriate. The other condition is the examination and diagnosis found that the disease is not serious, and only need to take outpatient care, so this admission is inappropriate. This suggests that the appropriateness of admission can change during hospitalization process.

To assess changes in the appropriateness of admission is valuable in reducing inappropriate admission and controlling the waste of medical resources, meanwhile, utilizing medical resources effectively so that diseases deserving hospitalization can be treated. Therefore, this study aims to analyze the admission behavior from a more comprehensive perspective, which explores the change in the appropriateness of admission after patients were admitted and its determinants, and provide a reference for physicians to make comprehensive and scientific medical decisions for admission.

## 2. Methods

### 2.1. Setting and participants

Counties and county-level cities in China's central (e.g. Dingyuan in Anhui Province) and western (e.g., Huining and Weiyuan in Gansu Province and Yilong in Sichuan Province) regions were designated as sample areas. The largest and most capable public comprehensive hospital in each sample county was selected as a sample hospital. The reimbursement and payment levels of the primary medical insurance, which may be associated with medical costs, as well as hospitalization behaviors (10), in the four counties are similar.

### 2.2. Sampling and data collection

Retrospective study was conducted to evaluate the appropriateness of admission. To study the changes in the appropriateness of admission, the evaluation of medical records is divided into two parts. One is to evaluate the appropriateness of admission according to the “admission records” in medical records. Admission records is a part of medical records, including identification, chief complaint, history of present illness, past history, family history, marital history, general physical exam, etc., which can reflect the patient's condition at the time of admission. The second is according the progress note, auxiliary examinations, doctor's orders and prescriptions, etc., in the medical records other than the “admission records” to evaluated the appropriateness of admissions which were defined as “inappropriate” in first part evaluation.

**Figure 1:**
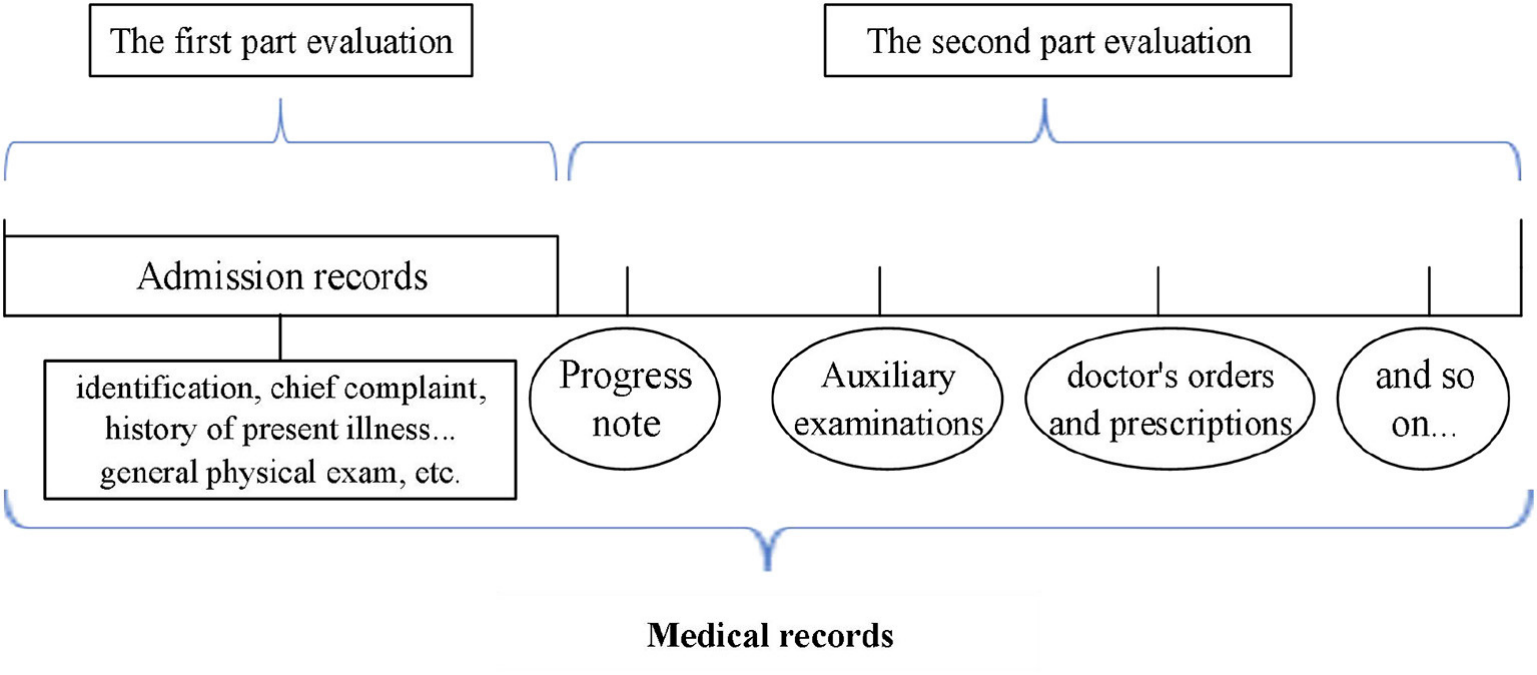
Schematic diagram of the admission records and medical records.

The progress note, auxiliary examinations, doctor's orders and prescriptions, etc., can reflect the development of the disease. Combined with the evaluation results of the two parts to explore the changes of the appropriateness of admission (shown in Figure 1).

In accordance with the existing research (11), the estimated inappropriate admission rate *P* is 16%, relative tolerance δ is 0.07, absolute tolerance d is 0.07^*^16% = 1.12%, the significance level α=0.05, and the one-sided standard normal deviation Zα =1.96. The equation of sample size (N) is as follows:


(1) $$\begin{array}{l} \begin{array}{lll} N\; & = & \;{\left(Z\alpha /d\right)}^{2}\;\times \;P\;(1\;-\;P)\;=\;{\left(1.96/1.12\%\right)}^{2}\;\times \;16\% \end{array}\\ \;\begin{array}{ll} \times & \;(1\;-\;16\%)\;=\;4,116 \end{array} \end{array}$$

**Figure 2:**
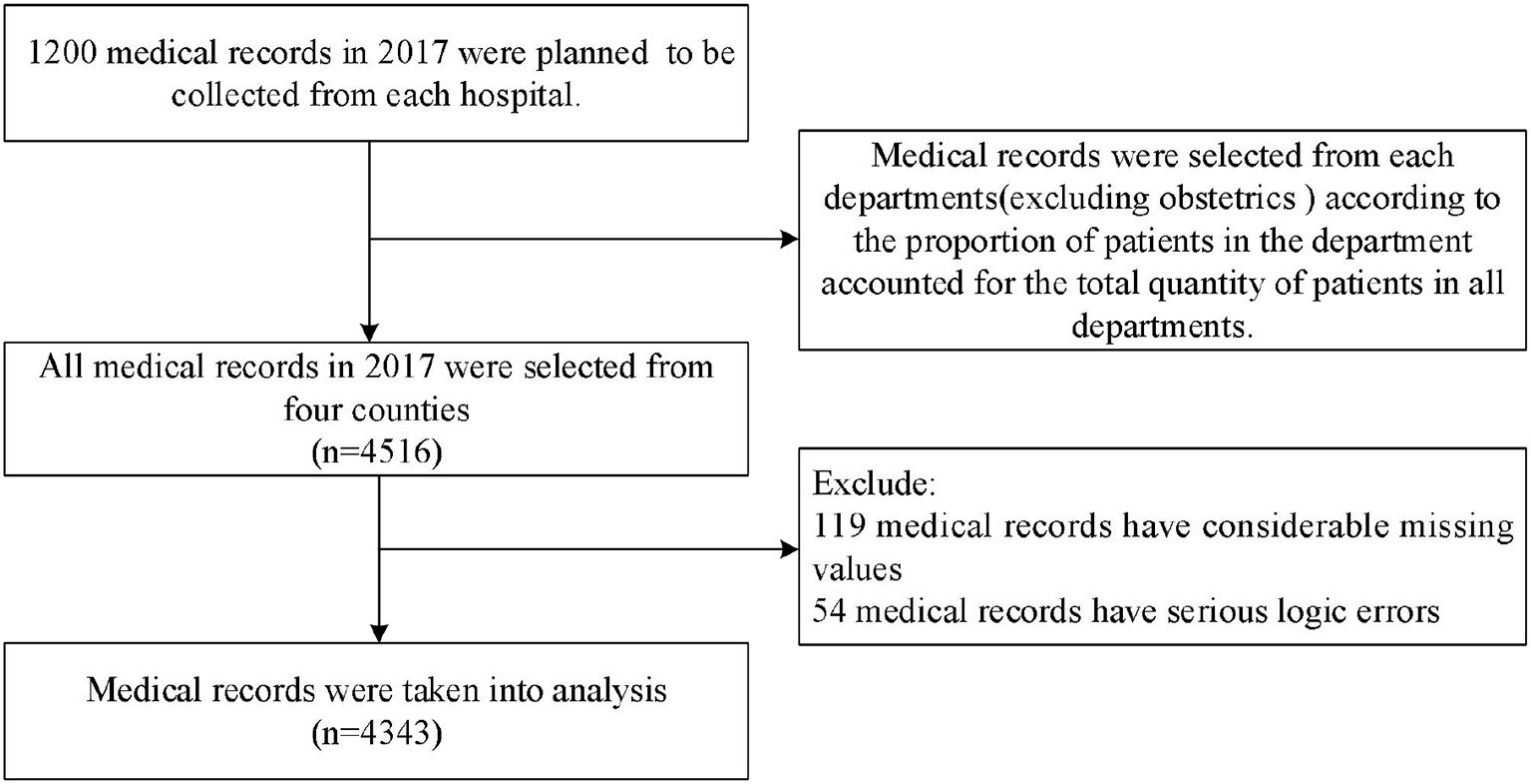
Study design and flow chart of the medical records selection.

Considering the quality of medical records, 1,200 medical records in 2017 were planned to be collected from each hospital. Firstly, admission for delivery is necessary and appropriate, and the services required for these cases are reasonable, so these records in obstetrics department were excluded considering the pertinence of AEP. Then, the corresponding quantity of medical records was selected from the remaining medical service departments in accordance with the proportion of patients in the department accounted for the total quantity of patients in all departments. After excluding medical records that had too many missing values and serious logic error, 4,343 medical records were taken into analysis (shown in Figure 2).

### 2.3. AEP and evaluation

AEP was originally developed by Gertman and Restuccia of the medical research center at Boston University school of medicine (18). A total of 16 criteria were used to evaluate the appropriateness of admission, including severity of illness and service intensity. Based on the AEP criteria of the United States, many countries have explored different AEP criteria that meet their own conditions (19, 20). The AEP criteria for county hospitals in China has been developed in 2014 (Supplementary Table for details) (10).

All medical records were evaluated by AEP criteria for county hospitals in this study. The medical records were evaluated by two trained experts. These highly trained experts involved in health service and policy research more than 5 years, all of whom received Ph.D. degrees and committed to making fair judgments on records in a strictly standard manner. They assessed the appropriateness of each medical records independently. The admission was considered appropriate if an actual value in medical record corresponded to the standard value in AEP. If no values in medical record conform to the AEP criteria, the admission was inappropriate. In other words, if any single one of the criteria was met, it would indicate that admission was appropriate. If no one of the criteria was met, would indicate that admission was inappropriate. Therefore, if a case was judged to be appropriate on the basis of admission records, the result of the admission was appropriate.

Each medical record was evaluated by each expert on the basis of the admission records and the rest part of medical records. The records that had different evaluation results between the two experts were judged by a third party, usually clinical experts. At last, reasonable evaluated results for admission record and the rest part of medical records were chosen after a comprehensive evaluation. Then, comparing two results reveals changes in admission appropriateness.

### 2.4. Statistics analysis

The diseases were categorized by using the International Classification of Diseases 10th revision (ICD-10) and were classified into several common disease categories. Age was divided into five groups according to children (0–14 years old), adolescents (15–19 years old), young adults (20–39 years old), the middle-aged (40–59 years old) and the aged (more than 59 years old). The medical services were medical, surgical, gynaecologic and pediatric. The severity of the patient upon admission, which were classified as “general, urgent, serious, and dangerous”.

The results of the admission appropriateness evaluation judged by the two parts were compared by McNemar's test. The binary logistic regression model was used to identify the determinants of changes in admission appropriateness. The dependent variable in the regression model was a binary variable, that is, “changed to appropriate or not” (“Yes” = 1, “No” = 0). The independent variables were first identified based on previous studies and whether could be obtained from the medical records, which included: Gender, Age, Type of medical insurance, Frequency of hospitalization, Medical service, Severity of the patient upon admission, History of disease, Having more than one disease, Disease category, With chronic disease. Then forward stepwise method was used to select independent variables in the final regression model (21).

The regression model is as follows:


(2) $$\begin{array}{l} \text{logit(P)}=\beta 0+\;\beta 1^{*}\text{Gender }+\;\beta 2^{*}\text{Age}\\ \;+\beta 3^{*}\text{Type of medical insurance}\\ \;+\beta 4^{*}\text{Frequency of hospitalization }\\ \;+\beta 5^{*}\text{Medical service}\\ \;+\beta 6^{*}\text{Severity of the patient upon admission}\\ \;+\beta 7^{*}\text{History of disease }\\ \;+\beta 8^{*}(\text{Having more than one disease})\;\\ \;+\beta 9^{*}\text{Disease category }\\ \;+\beta 10^{*}\text{With chronic disease} \end{array}$$

The statistical analysis was performed by IBM SPSS Statistics 20.0. *P* < 0.05 was considered statistically significant.

## 3. Results

### 3.1. The evaluation results of the admission records and the rest part of medical records

As shown in Table 1, by evaluating admission records, 942 (21.69%) records were appropriate, 3,401 (78.31%) records were inappropriate. Judging the 3,401 records again according to the rest part of the medical records, it was concluded that 2,224 of the 3,401 inappropriate admissions changed to appropriate, accounting for 65.39%. 1,177 cases were still inappropriate, accounting for 34.61%. A total of 3,166 records were judged as appropriate admissions, and 1,177 records were judged as inappropriate admissions. Inappropriate admission rate was 27.1%. The difference between the two parts evaluation was statistically significant (*P* < 0.001).

**Table 1:** Appropriateness evaluation of the admission records and the rest part of medical records.

**The admission records**	**The rest part of medical records**		**Total**
	**Appropriate** ***N*** **(%)**	**Inappropriate** ***N*** **(%)**	
Appropriate	942 (100.00)	0 (0.00)	942
Inappropriate	2,224 (65.39)	1,177 (34.61)	3,401
Total	3,166 (72.90)	1,177 (27.10)	4,343

### 3.2. Characteristics of patients that inappropriate at admission but appropriate at discharge

As shown in Table 2, 65.39% of the 3,401 inappropriate admissions changed to appropriate finally. The appropriate rate of females (67.1%, *P* = 0.043) was higher than that of males (63.8%, *P* = 0.043). The highest appropriate rate was found among the older adult (74.4%, *P* < 0.001), whereas the lowest was found among children (45.3%, *P* < 0.001). Inpatients with “medical assistance” had higher appropriate rate (84.8%, *P* < 0.001) than inpatients with new rural cooperative medical scheme (NRCMS) and “medical insurance for urban residents and workers”. Inpatients in Medical (69.1%, *P* < 0.001) and Gynaecologic (72.8%, *P* < 0.001) had high appropriate rate. Inpatients who had “serious” status upon admission (70.2%, *P* < 0.001), who had more than one disease (76.8%, *P* = 0.006) and who with chronic diseases (74.1%, *P* < 0.001) were prone to get appropriate finally. Inpatients suffering from urinary diseases (81.9%, *P* < 0.001), genital diseases (80.3%, *P* < 0.001) and circulatory diseases (77.3%, *P* < 0.001) had the highest appropriate rate, whereas those suffering from respiratory diseases had the lowest (44.2%, *P* < 0.001).

**Table 2:** Distribution of characteristics of patients that evaluated as inappropriate at admission (*n* = 3.401).

**Variable**	**All (column %)**	**Changed to appropriate at discharge**		***P*-value**
		**Yes**	**No**	
		**Number (%)**	**Number (%)**	
Total	3,401 (100.00)	2,224 (65.39)	1,177 (34.61)	
**Sex**				0.043
Male	1,745 (51.31)	1,113 (63.80)	632 (36.20)	
Female	1,656 (48.69)	1,111 (67.10)	545 (32.90)	
**Age**				< 0.001
0–14	664 (19.52)	301 (45.30)	363 (54.70)	
15–19	102 (3.00)	66 (64.70)	36 (35.30)	
20–39	362 (10.64)	240 (66.30)	122 (33.70)	
40–59	974 (28.64)	650 (66.70)	324 (33.30)	
More than 59	1,299 (38.19)	967 (74.40)	332 (25.60)	
**Type of medical insurance**				< 0.001
NRCMS	1,566 (46.05)	1,011 (64.60)	555 (35.40)	
Medical insurance for urban residents	778 (22.88)	422 (54.20)	356 (45.80)	
Medical insurance for urban workers	521 (15.32)	379 (72.70)	142 (27.30)	
Medical assistance	363 (10.67)	308 (84.80)	55 (15.20)	
Commercial health insurance	39 (1.15)	24 (61.50)	15 (38.50)	
Self-payment	119 (3.50)	65 (54.60)	54 (45.40)	
Others	15 (0.44)	15 (100.00)	0 (0.00)	
**Frequency of hospitalization**				0.069
Once	3,240 (95.27)	2,108 (65.10)	1,132 (34.90)	
More than once	161 (4.73)	116 (72.00)	45 (28.00)	
**Medical service**				< 0.001
Pediatric	538 (15.82)	250 (46.50)	288 (53.50)	
Medical	1,323 (38.90)	914 (69.10)	409 (30.90)	
Surgical	836 (24.58)	542 (64.80)	294 (35.20)	
Gynaecologic	202 (5.94)	147 (72.80)	55 (27.20)	
Others	502 (14.76)	371 (73.90)	131 (26.10)	
**Severity of the patient upon admission**				< 0.001
General	540 (15.88)	352 (65.20)	188 (34.80)	
Urgent	2,329 (68.48)	1,555 (66.80)	774 (33.20)	
Serious	285 (8.38)	200 (70.20)	85 (29.80)	
Dangerous	247 (7.26)	117 (47.40)	130 (52.60)	
**History of diseases**				< 0.001
No	2,580 (75.86)	1,642 (63.60)	938 (36.40)	
Yes	821 (24.14)	582 (70.90)	239 (29.10)	
**Having more than one disease**				0.006
No	3,276 (96.32)	2,128 (65.00)	1,148 (35.00)	
Yes	125 (3.68)	96 (76.80)	29 (23.20)	
**Disease category**				< 0.001
Circulatory diseases	546 (16.05)	422 (77.30)	124 (22.70)	
Injury and poisoning	188 (5.53)	141 (75.00)	47 (25.00)	
Endocrine diseases	103 (3.03)	67 (65.00)	36 (35.00)	
Urinary diseases	127 (3.73)	104 (81.90)	23 (18.10)	
Respiratory diseases	1,049 (30.84)	464 (44.20)	585 (55.80)	
Digestive diseases	633 (18.61)	452 (71.40)	181 (28.60)	
Skeletal and muscular diseases	123 (3.62)	76 (61.80)	47 (38.20)	
Genital diseases	213 (6.26)	171 (80.30)	42 (19.70)	
Others	419 (12.32)	327 (78.00)	92 (22.00)	
**With chronic diseases**				< 0.001
No	2,806 (82.51)	1,783 (63.50)	1,023 (36.50)	
Yes	595 (17.49)	441 (74.10)	154 (25.90)	

NRCMS, new rural cooperative medical scheme. It was a type of primary health insurance for rural residents in China before integrated with medical insurance for urban residents.

### 3.3. Factors affecting the changes in the appropriateness of admission

As shown in Table 3, binary logistic regression analysis showed that age, type of medical insurance, medical service, severity of the patient upon admission and disease category were determinants of affecting the changes in the appropriateness of admission in county hospitals. Older patients (OR = 3.658, 95% CI: [2.462–5.435]; *P* < 0.001) were positively associated with changing from “inappropriate” to “appropriate” than younger patients. Inpatients covered by medical assistance (OR = 2.661, 95% CI: [1.926–3.676]; *P* < 0.001) were positively associated with being considered appropriate admissions at discharge than those covered by NRCMS. Self-payment (OR = 0.656, 95% CI: [0.434–0.993]; *P* = 0.046) had the lowest possibility be evaluated as appropriate at discharge. Compared with others, inpatients in the pediatric were positively associated with changing from “inappropriate” to “appropriate”. In terms of severity of the patient upon admission, urgent patients (OR = 1.392, 95% CI: [1.109–1.747]; *P* = 0.004) and serious patients (OR = 1.48, 95% CI: [1.041–2.105]; *P* = 0.029) were positively associated with changing from “inappropriate” to “appropriate” than ‘general' patients. They rarely occurred among the patients labeled as “dangerous” (OR = 0.593, 95% CI: [0.421–0.835]; *P* = 0.003). Compared with circulatory diseases, the case be evaluated as “appropriate” at discharge was positively associated with the urinary diseases (OR = 1.709, 95% CI: [1.019–2.865]; *P* = 0.042) and genital diseases (OR = 2.998, 95% CI: [1.737–5.174]; *P* < 0.001), whereas the opposite finding was observed for respiratory diseases (OR = 0.347, 95% CI: [0.268–0.451]; *P* < 0.001) and skeletal and muscular diseases (OR = 0.556, 95% CI: [0.355–0.873]; *P* = 0.011).

**Table 3:** Binary logistic regression analysis of the factors affecting the changes in the appropriateness of admission (*n* = 3,401).

**Characteristics**	**Adjusted OR**	**95% confidence interval of AOR**		***P*-value**
		**Lower limit**	**Upper limit**	
**Constant**	1.514			0.020
**Age (Ref. 0–14)**				< 0.001
15–19	1.868	1.071	3.258	0.028
20–39	2.045	1.325	3.156	0.001
40–59	2.146	1.449	3.179	< 0.001
more than 59	3.658	2.462	5.435	< 0.001
**Type of medical insurance (Ref. NRCMS)**				< 0.001
Medical insurance for urban residents	0.616	0.503	0.755	< 0.001
Medical insurance for urban workers	1.285	1.01	1.634	0.041
Medical assistance	2.661	1.926	3.676	< 0.001
Commercial health insurance	0.77	0.387	1.534	0.458
Self-payment	0.656	0.434	0.993	0.046
**Medical service (Ref. Pediatric)**				< 0.001
Medical	0.548	0.356	0.844	0.006
Surgical	0.422	0.279	0.636	< 0.001
Gynaecologic	0.337	0.177	0.644	0.001
Others	0.689	0.431	1.1	0.119
**Disease category (Ref. circulatory diseases)**				< 0.001
Injury and poisoning	1.507	0.973	2.334	0.066
Endocrine diseases	0.771	0.48	1.238	0.281
Urinary diseases	1.709	1.019	2.865	0.042
Respiratory diseases	0.347	0.268	0.451	< 0.001
Digestive diseases	1.144	0.849	1.542	0.378
Skeletal and muscular diseases	0.556	0.355	0.873	0.011
Genital diseases	2.998	1.737	5.174	< 0.001
Others	1.668	1.191	2.335	0.003
**Severity of the patient upon admission (Ref. general)**				< 0.001
Urgent	1.392	1.109	1.747	0.004
Serious	1.48	1.041	2.105	0.029
Dangerous	0.593	0.421	0.835	0.003

AOR, adjusted odds ratio.

## 4. Discussion

Through the descriptive analysis and logistic regression analysis, we investigated the situations and factors associated with the changes in the appropriateness of admission in county hospitals in rural China. According to the results of the study, we further analyzed the causes of the changes in the appropriateness of admission from two aspects of individual characteristics and disease characteristics of inpatients.

### 4.1. Changes in the appropriateness of admission after the patients was admitted

As so far, this study is the first one to concern changes in the appropriateness of admission after the patients was admitted in county hospitals in rural China. The study showed that 65.39% of the inappropriate cases at admission changed to appropriate at discharge. The result may be attributed to the following reasons. First, the admission appropriateness evaluation when the patients is admitted is based on the patients' indications before and at the time of admission. However, diseases are complex and have insidious characteristics, and change constantly (22). In addition, different patients have different symptoms, severity and different development stages of diseases at the time of admission. Some patients are in the early stage of disease and their disease characteristics does not fully emerge. In other words, the patients may not meet the admission criteria at the time of admission but their conditions might get serious after admission. The risk of deterioration and vulnerabilities of some patients increase the uncertainty of their conditions (23). All of this make the admission appropriateness evaluation when patients were admitted less comprehensive and accurate. Second, as an evaluation instrument, the AEP cannot fully substitute for the professional judgments of clinicians (24), which results in the appropriateness assessment of admissions lack of flexibility (18, 25).

### 4.2. Individual characteristics of patients that evaluated as inappropriate at admission but appropriate at discharge

The study found that older patients' admissions were more likely to go from “inappropriate” to “appropriate” than younger patients. This finding may be attributed to older patients have multiple types of diseases occurring simultaneously, majority of which are chronic diseases. Insidious onset and clinical symptoms are not typical but start to emerge after admission (26, 27). However, younger patients are mainly affected by acute diseases, which are easily identified (28, 29). Given that the middle-aged and older adult patients constitute the majority (69.7%) in medical assistance, inpatients are easily judged to be appropriate admissions at discharge compared with the NRCMS inpatients (30, 31). The admission of patients with medical insurance for urban residents is not easy change to appropriate, indicating that compared with rural inpatients, urban residents are prone to use hospital services unreasonably, while urban workers are on the contrary (32, 33).

### 4.3. Disease characteristics of patients that evaluated as inappropriate at admission but appropriate at discharge

As one of the special groups, children had different characteristics compared with adults. The function of children's various organs will develop with age. For the same pathogenic factor, there are considerable differences in the pathological reaction and disease development between children and adults (34). For example, pneumonia caused by pneumococcus is more common in infants with bronchopneumonia, while lobar pneumonia may be present in adults and older children (35, 36). The types and clinical manifestations of childhood diseases are quite different from that of adults. Therefore, there may be deviation in using the same set of criteria to measure the admission appropriateness for special population. Inpatients in the pediatric are easily categorized as inappropriate in admission but they were appropriate admission actually. This suggests that the admission recognition criteria applicable to general patients may not be suitable for pediatrics. It is necessary to add key indicators that meet the characteristics of pediatric patients and further demonstrate and test in practice.

This study showed that patients with “dangerous” status upon admission are not likely to go from inappropriate to appropriate. Patients with “dangerous” status upon admission are more likely to reach the admission criteria than patients with general status theoretically. Patients who are admitted in a “dangerous” condition are easily rated as appropriate admission. The “dangerous” cases were rated as inappropriate may be related to doctors' judgment bias at the time of consultation and the nonstandard writing of medical records. The circulatory disease, namely cardiovascular and cerebrovascular diseases have very complex symptoms and conditions (37, 38). Due to the diversity of symptoms and the logicality of the patient's description varies greatly, the development degree of the disease cannot be clearly determined by the self-reported or other-mentioned symptoms nor the “sight, touch, knock and listen” of physical examination (39–41). The criteria of pulse and blood pressure in AEP cannot cover the signs of circulatory disease. The patients who are at risk for underlying diseases were more likely to reach the admission criteria at discharge. The urinary and genital diseases are mostly caused by infection because of the particularity of the diseases themselves (42). Mycoplasma is one of the pathogens causing urinary and genital infections. However, due to its small size, it grows slowly in the medium and is difficult to be observed, so the clinical diagnosis of mycoplasma infection is more difficult (43, 44). So, it is difficult to determine the severity of the urinary and genital diseases at the time of admission accurately. Nevertheless, respiratory diseases can be diagnosed by listening to breathing sounds, and the skeletal and muscular diseases can be easily diagnosed through viewing and touching (45, 46). The external characteristics of these two diseases are relatively obvious, and it is very easy to be diagnosed at the time of admission. The probability of going from inappropriate to appropriate is lower compared to circulatory, urinary and genital diseases.

## 5. Limitations

This study has several limitations. Firstly, although our study identified the determinants that affecting the change in the appropriateness of admission, a causal inference was not identified because of the cross-sectional research design. Secondly, the indicators in medical records cannot fully reflect patients' conditions during admission, and the characteristics of physicians cannot be extracted from the medical records, physicians characteristics were not included in the analysis. In addition, medical records may not be completely accurate because of the lack of rules and regulations for medical records, which may affect the results of admission evaluation.

## 6. Conclusions

Nearly two-in-thirds (65.39%) of the 3,401 inappropriate admissions changed to appropriate finally. It is noticing that there were still 34.61% of the 3,401 inappropriate admissions remaining inappropriate, which needs to be strictly controlled. The indicators, such as age, type of medical insurance, medical service, severity of the patient upon admission and disease category, were found to be closely associated with the changes in the appropriateness of admission in county hospitals. On one hand, the evaluation indicators of AEP need further improvement. On the other hand, physicians and regulators need to take a dynamic view of disease progression and inappropriate admission. Aside from referring to AEP criteria, physicians and evaluators both should pay attention to individual and disease characteristics to make a comprehensive judgment, with specific attention paid to the older and the patients suffering from the urinary, genital and circulatory diseases, so that patients who need inpatient services get the necessary hospital treatments. At the same time, strict control and attention should be paid to the admission of respiratory diseases and skeletal and muscular diseases. Regulators should give a flexible range of “inappropriate admission rates” and evaluated the appropriateness of admission comprehensively to reduce judgment errors and patients' disease risks.

## Data availability statement

The raw data supporting the conclusions of this article will be made available by the authors, without undue reservation.

## Author contributions

JC and HL made substantial contributions to the conception, study design, analyses, and writing of the manuscript. YC and HG contributed to draft, review, and revise the manuscript. DS contributed to the data acquisition and provided statistical analysis support. JC drafted the article. All authors supplied critical revisions to the manuscript and gave final approval of the version to be published.
