# Supplementary material for: Is there a change in the appropriateness of admission after patients were admitted? Evidence from four county hospitals in rural China
Source: Front Public Health. 2023 May 25;11:1106499. doi: 10.3389/fpubh.2023.1106499 (PMC10248166; doi:10.3389/fpubh.2023.1106499)
Supplement: Supplementary file 1 [file Table_1.DOCX]

Supplementary Material

Is there a change in the appropriateness of admission after patients were admitted? Evidence from four county hospitals in rural China

**Jingjing Chang^1^, Hongxia Gao****^2,3^, Dai Su^4^, Haomiao Li^5*^, Yingchun Chen^2,3,6*^**

^1^Wuhan Children's Hospital (Wuhan Maternal and Child Healthcare Hospital), Tongji Medical College, Huazhong University of Science & Technology, Wuhan, China;

^2^ School of Medicine and Health Management, Tongji Medical College, Huazhong University of Science and Technology, Wuhan,China;

^3^ Research center for Rural Health Services, Hubei Province Key Research Institute of Humanities and Social Sciences, Wuhan, China;

^4^ School of Public Health, Capital Medical University, Beijing, China;

^5^ School of Political Science and Public Administration, Wuhan University, Wuhan, China;

^6^ Lead contact.

*** Correspondence:**

Yingchun Chen: [chenyingchunhust@163.com](mailto:chenyingchunhust@163.com)

Haomiao Li: [lihaomiao@whu.edu.cn](mailto:lihaomiao@whu.edu.cn)

#

# Supplementary Table **AEP criteria for county hospitals.**

| **A. Medical service intensity** |
| --- |
| A1. Need follow-up treatment within 24 h: (1) instruments or other facilities that are only available for hospitalised patients (angiography, visceral biopsy, cardiac catheterisation intervention) and/or (2) invasive diagnostic of central skeletal muscle meat (lumbar puncture, cisterna puncture, ventricular puncture, encephalography) |
| A2. Treatment with varying dosages or drugs on a regular basis under direct medical supervision |
| A3. Calculation of intake and output volume |
| A4. Operation to be conducted on the following day in the operating room, detailed pre-operative consultation or evaluation on the day of admission |
| A5. Main surgical incision and drainage nursing |
| A6. Quarantined patients |
| A7. Bedside electrocardiogram (ECG) monitoring or testing vital signs at least every 2 h |
| A8. Stopping (at least once every 8 h) or continuing oxygen inhalation |
| A9. Referral of post-operative recovery |
| **B. Disease severity** |
| B1. Continuous fever > 38.0 °C for more than 5 days |
| B2. Acute confusion (coma or adiaphoria) |
| B3. Severe anomaly in electrolyte or blood and vigour, showing the following situations: (1) Na < 123 mEq/L or > 156 mEq/L; (2) K < 2.5 mEqt/L or > 6.0 mEq/L; (3) HCO_3_ < 20 mEq/L or > 36 mEq/L; and (4) arterial blood pH < 7.30 or > 7.45 |
| B4. Loss of sight or hearing for 48 h |
| B5. Loss of activity in any part of the body for 48 h |
| B6. Excretion disorder or absence of intestinal peristalsis in the past 24 h |
| B7. Active bleeding |
| B8. Needing blood transfusion because of bleeding |
| B9. Mental disorders caused by non-alcohol dependence |
| B10. Viscera removal or surgical wound dehiscence |
| B11. Pulse less than 50 or greater than 140 beats per minute |
| B12. Abnormal blood pressure: systolic blood pressure < 90 mmHg or > 200 mmHg and/or diastolic blood pressure < 60 mmHg or > 120 mmHg |
| B13. Ventricular fibrillation or acute myocardial ischemia shown by electrocardiogram (ECG) report or course log |
| B14. Acute hematopathy, severe medium-sized leukopenia, thrombocytopaenia, leukocytosis, erythrocytosis, thrombocytosis or haemolysis-resulted symptoms |
| B15. Progressive acute neurological disorders |
| B16. Soft tissue injuries affecting basic self-care |
| B17. Acute myocardial infarction or cerebrovascular accident (stroke) |
| B18. Spinal cord lesions |
| B19. Lung infection above 50% or leafy lesions according to X-ray examination |
| B20. Hyperemesis or acute pain caused by acute or chronic diseases |

**Note:** The AEP criteria for the county hospital was derived from the research results of a National Natural Science Foundation project undertaken by our research team. *(《Research on Measurement and Management of Excessive Demand for Inpatient Service of New Rural Cooperative Medical Scheme》, NO. 71073061)*. It was based on the experience of international AEP criteria, and was combined with the reality of rural China. After several rounds of expert consultation and combined with field research, the AEP criteria for county hospital was accomplished and a monograph (Excessive demand for rural hospitalization service—a study on the measurement and management of inappropriate admission) has been published.
